# A Robust and Wearable Triboelectric Tactile Patch as Intelligent Human-Machine Interface

**DOI:** 10.3390/ma14216366

**Published:** 2021-10-24

**Authors:** Zhiyuan Hu, Junpeng Wang, Yan Wang, Chuan Wang, Yawei Wang, Ziyi Zhang, Peng Xu, Tiancong Zhao, Yu Luan, Chang Liu, Lin Qiao, Mingrui Shu, Jianchun Mi, Xinxiang Pan, Minyi Xu

**Affiliations:** 1 Dalian Key Laboratory of Marine Micro/Nano Energy and Self-Powered Systems, Marine Engineering College, Dalian Maritime University, Dalian 116026, China; zhiyuanhu@dlmu.edu.cn (Z.H.); wangjp@dlmu.edu.cn (J.W.); wangyanme@dlmu.edu.cn (Y.W.); labrador@dlmu.edu.cn (C.W.); wangyawei@dlmu.edu.cn (Y.W.); scarletziy@dlmu.edu.cn (Z.Z.); pengxu@dlmu.edu.cn (P.X.); theboyismute_@dlmu.edu.cn (Y.L.); dudu@dlmu.edu.cn (C.L.); 2 Center for Intelligent Sensors and MEMS (CISM), National University of Singapore, Singapore 117576, Singapore; 3 School of Marine Engineering and Technology, Sun Yat-sen University, Guangzhou 510275, China; zhaotc3@mail2.sysu.edu.cn; 4 Navigation College, Dalian Maritime University, Dalian 116026, China; qiao@dlmu.edu.cn; 5 Institute for Ocean Engineering, Tsinghua Shenzhen International Graduate School, Shenzhen 518000, China; smr20@mails.tsinghua.edu.cn; 6 College of Engineering, Peking University, Beijing 100871, China; jcmi@coe.pku.edu.cn; 7 School of Electronics and Information Technology, Guangdong Ocean University, Zhanjiang 524088, China; dmupanxx@gmail.com

**Keywords:** triboelectric nanogenerator, hydrogels, tactile patch, human–machine interface, robot control

## Abstract

The human–machine interface plays an important role in the diversified interactions between humans and machines, especially by swaping information exchange between human and machine operations. Considering the high wearable compatibility and self-powered capability, triboelectric-based interfaces have attracted increasing attention. Herein, this work developed a minimalist and stable interacting patch with the function of sensing and robot controlling based on triboelectric nanogenerator. This robust and wearable patch is composed of several flexible materials, namely polytetrafluoroethylene (PTFE), nylon, hydrogels electrode, and silicone rubber substrate. A signal-processing circuit was used in this patch to convert the sensor signal into a more stable signal (the deviation within 0.1 V), which provides a more effective method for sensing and robot control in a wireless way. Thus, the device can be used to control the movement of robots in real-time and exhibits a good stable performance. A specific algorithm was used in this patch to convert the 1D serial number into a 2D coordinate system, so that the click of the finger can be converted into a sliding track, so as to achieve the trajectory generation of a robot in a wireless way. It is believed that the device-based human–machine interaction with minimalist design has great potential in applications for contact perception, 2D control, robotics, and wearable electronics.

## 1. Introduction

With the rapid development of the Internet of Things (IoT) and artificial intelligence (AI), various tactile human–machine interfaces (HMIs) have been developed in a wide range of applications [1,2,3]. Flexible and wearable sensors have been developed widely because they can be easily integrated with HMIs compared with traditional rigid sensors. Recently, HMIs based on flexible and wearable sensors have been applied in the fields such as electro-tactile stimulation, motion recognition, flexible displays, robot control, healthcare [4], and virtual reality/augmented reality (VR/AR) [5,6,7]. For example, people can directly interact with mobile phones and smart furnitures by using their voice. In addition, it is possible to control the machine by detecting people’s intentions through human motions. The self-powered function is very important for the distributed HMIs system to reduce the overall power consumption of the entire system and achieve sustainable operation without battery. The transducing mechanisms of common sensors can be divided into resistive [8,9], capacitive [10,11], piezoelectric [12,13], and triboelectric [14,15]. An external power source is essential for the resistive and capacitive sensors. Piezoelectric and triboelectric electronic devices can generate signals in response to external mechanical stimuli, which are ideal to construct self-powered interfaces.

Since 2012, a technology of triboelectric nanogenerator (TENG) has been developed based on the coupling effect of contact electrification and electrostatic induction. It has received great attention worldwide due to its high output performance, wide range of material availability, flexibility, and ductility [16]. It can be used as both various types of self-powered sensors and energy harvesters. The TENG has been used for various kinds of energy harvesting [17,18,19,20,21,22], self-powered sensors [23,24,25,26,27], self-powered healthcare detections [28,29,30], and other fields at present. Due to a variety of flexible materials used in TENG recently, TENG-based flexible and wearable sensors were proposed for tactile perception [31,32,33,34,35], intelligent human–machine interaction [36,37,38], and electronic skin [39,40]. Flexible and wearable sensors are critical to build up HMI systems with self-powered supply. Various wearable, self-powered HMIs system through finger touching and sliding have been extensively studied recently. Yi et al. [41] produced a flexible 26-units sensor array to construct a self-powered keyboard; the device was further sewed into a large-area pressure sensor array as a self-powered wearable keyboard. The device has biological recognition function, which can identify the operation users. The self-powered wearable keyboards has practical applications in human–computer interaction devices and personal user identification systems. Tang et al. [42] reported a 7.2 cm × 7.2 cm flexible and wearable triboelectric sensor array with four channels for unmanned aerial vehicle (UAV) trajectories control. The UAV could perform a variety of actions through different voltage output ratios. Tao et al. [43] made a combination of 2D self-powered, flexible, triboelectric sensor (SFTS) patch and 1D SFTS patch based on different voltage output ratio to control the 3D movement of robot hands, providing a new control method for the robot in the industrial field. Some of these developed multi-sensing arrays of the common flexible and wearable TENG-based devices are able to control robot or computer. However, multi-channel TENG adopts a signal-combined method for robot controlling or sensoring. The signal combination caused the sensor signal change because the phase and amplitude of the sensor signal generated by TENG deviated, which is not conducive to the stablilty of sensing and control of the robot based on TENG.

Therefore, a new type of self-powered, minimalist, and more effective interactive devices is in high demand to provide good stability. A robust and wearable triboelectric patch (RWTP) based on TENG was developed and examined in the present work. RWTP is proposed to achieve HMIs, pressure sensing, or contact position perception through the multi-channel array. More specifically, to improve the stability of the sensor signal, this work applied hydrogel electrodes and a circuit for signal processing, which can keep the amplitude of the processed signal stable (the deviation of the amplitude within 0.1 V) and transfer the external force into the deformation of the RWTP. To detect the position of an individual touching and trace of a continuous sliding, each channel of RWTP is connected to the single-chip microcomputer I/O separately, and the position of the finger click is determined by the number of channels. Through the application of signal processing and hydrogels electrodes, RWTP as a wearable device has high biocompatibility. The signal processing circuit can be more stable to realize the human–machine interfaces between RWTP and the robot. RWTP can be used as a kind of human. The controlling of machine interaction is applied in the field of engineering, such as with industy robot. The combination of multi-channel control and TENG can be applied to human–machine interfaces. It can realize the control of robots in a minimalist way, which has deep application value in the industrial field. Further, a specific algorithm is used to convert the 1D serial number into a 2D coordinate system, so that the click of the finger can be converted into a sliding track, so as to achieve trajectory generation of robot in a wireless way.

## 2. Results and Discussions

**Figure 1:**
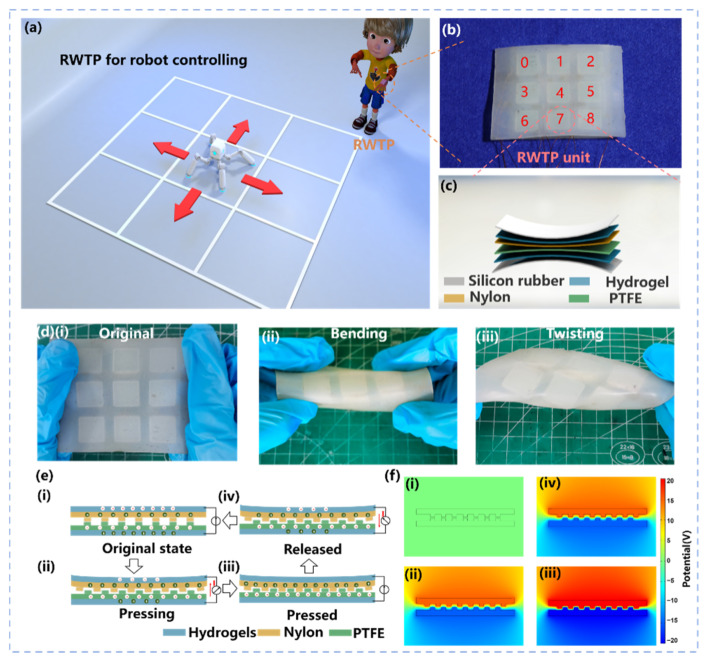
Structure design and working principle of RWTP: (**a**) schematic of real-time control of a robot; (**b**) image of an as-fabricated RWTP; (**c**) layer-by-layer structure of RWTP unit; (**d**) images of RWTP in its (**i**) original, (**ii**) bending, and (**iii**) twisting states; (**e**) working principle of RWTP; (**f**) potential simulations by COMSOL to elucidate the working principle (**i**) original state, (**ii**) pressing state (**iii**) pressed state and (**iv**) released state.

**Figure A1:**
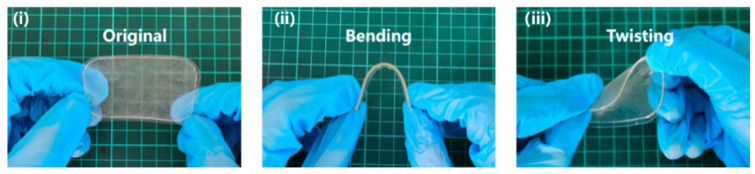
Flexibility diagram of hydrogels electrodes. (**i**) original state of hydrogel (**ii**) bending state of hydrogel (**iii**) twisting state of hydrogel.

An application scenario of the wearable robot controlling patch is shown in Figure 1a, where the RWTP is attached to the arm. When the executor makes an operation on the RWTP, the generated electrical signals from the patch of each step can be acquired and sent to the robot. The RWTP consists of nine controlling units as shown in Figure 1b. The RWTP with a total size of. The detailed controlling unit is presented in Figure 1c, which is fabricated by a flexible silicone rubber substrate, a tribo-layer and two hydrogel electrodes. To enhance the contact electrification, nylon and polytetrafluoroethylene (PTFE) are used as the tribo-layer. Nylon and PTFE are two dielectric materials with different ability of losing and gaining electrons. After being forced into contact with each other, the inner surfaces of the two triboelectric layers will have opposite static charges (tribo-charges) with equal density as a result of contact electrification. Different from the solid electrodes, hydrogel electrodes can be more effective to transfer the external force into deformation of RWTP because of the high flexibility of hydrogels (Appendix A Figure A1). Therefore, as shown in Figure 1d(i–iii), the RWTP displays flexibility after a series of intense deformations, including twisting and bending. The theoretical equation for representing the real-time power generation of a TENG is a relationship among three parameters: the output voltage (*V*) between the two electrodes, the amount of transferred charge (*Q*) in between, and the separation distance (*x*) between the two triboelectric layers, which can be named the *V–Q–x* relationship. At the surface of these two dielectrics, two hydrogel layers are used as two electrodes. After being forced to make contact with each other, the two triboelectric layers will have opposite static charges (tribocharges), with equal density of σ, as a result of contact electrification. From the Gauss theorem, the electric field strength at each region is given by the following [44]:

(1) $$\mathrm{Inside}\;\mathrm{Dielectric}\;1:E_{1}=-\frac{Q}{S\varepsilon_{0\;}\varepsilon_{1}}\;$$

(2) $$\mathrm{Inside}\;\mathrm{the}\;\mathrm{air}\;\mathrm{gap}:E_{air}=-\frac{Q}{S\varepsilon_{0}}+\frac{\sigma x\left(t\right)}{\varepsilon_{0}}\;$$

(3) $$\mathrm{Inside}\;\mathrm{Dielectric}\;2::E_{2}=-\frac{Q}{S\varepsilon_{0\;}\varepsilon_{2}}$$

where *S* is the contact area of two dielectric materials, and $\varepsilon_{0}$ is the relative dielectric constants in the air. Two dielectric materials (nylon and PTFE), with thicknesses of $d_{1}$ and $d_{2},$ and the relative dielectric constants $\varepsilon_{r1}$ and $\varepsilon_{r2}$ , respectively, are stacked face-to-face as two triboelectric layers. The output voltage between the two electrodes can be given by the following:

(4) $$V=E_{1}d_{1}+E_{air}x+E_{2}d_{2}$$

Substituting Equations (1)–(3) into Equation (4) leads to the following *V*–*Q*–*x* relationship for the RWTP in contact mode:

(5) $$V=-\frac{Q}{S\varepsilon_{0}}\left(\frac{d_{1}}{\varepsilon_{r1}}+\frac{d_{2}}{\varepsilon_{r2}}+x\left(t\right)\right)+\frac{\sigma x\left(t\right)}{\varepsilon_{0}}$$

In Equation (5), $S,d_{1},d_{2},$ and x are those variables which determine the output voltage of the RWTP. The thicknesses of two dielectric materials, $d_{1}\;\mathrm{and}\;d_{2}$ , can be approximated as a constant in RWTP. The influence of *S* and separation distance *x* of RWTP on the output voltage are shown later.

The working principle of the RWTP and its electrical performance are shown in Figure 1e(i–iv). As shown in the Figure 1e(i–iv), when an external force is applied, two triboelectric layers are brought into contact with each other, and the contact area that related to external forces is changed. Surface-charge transfer then takes place at the contact area due to triboelectrification effect. The increasing force leads to increasing contact area of Nylon and PTFE, and more transferred charges are generated on the triboelectric layers. Therefore, the current increases with the increase of force.

**Figure A2:**
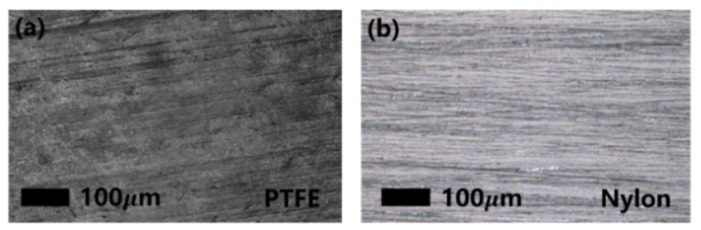
Microstructure of nylon and PTFE. (**a**) microstructure of PTFE (**b**) microstructure of Nylon.

In order to verify the working principle of Figure 1d, the surface microstructures of two dielectric materials were photographed (see Appendix A Figure A2). When an external force is applied, two tribo-layers make contact with each other, and the contact area related to the external force changes. Surface-charge transfer occurs in the contact area due to the electrostatic induction. Electrons are transferred from the PTFE hydrogel to the nylon hydrogel (Figure 1e(i–ii). When the external force is released, electrons will flow back from nylon hydrogel to PTFE hydrogel. Thus, stable voltage and alternating current signals can be generated by external force (Figure 1e(iii,iv). To clearly demonstrate the working principle of the RWTP, the electric potential distributions of the corresponding states were simulated by COMSOL multiphysics software (COMSOL 5.6). Models used in the COMSOL simulation software are Alternating current/Direct current modules. The instantaneous electric field of the RWTP is calculated, and the geometric parameters of the simulated model are the same as the real device. The full cycle of the potential changes is shown in Figure 1f(i–iv).

**Figure 2:**
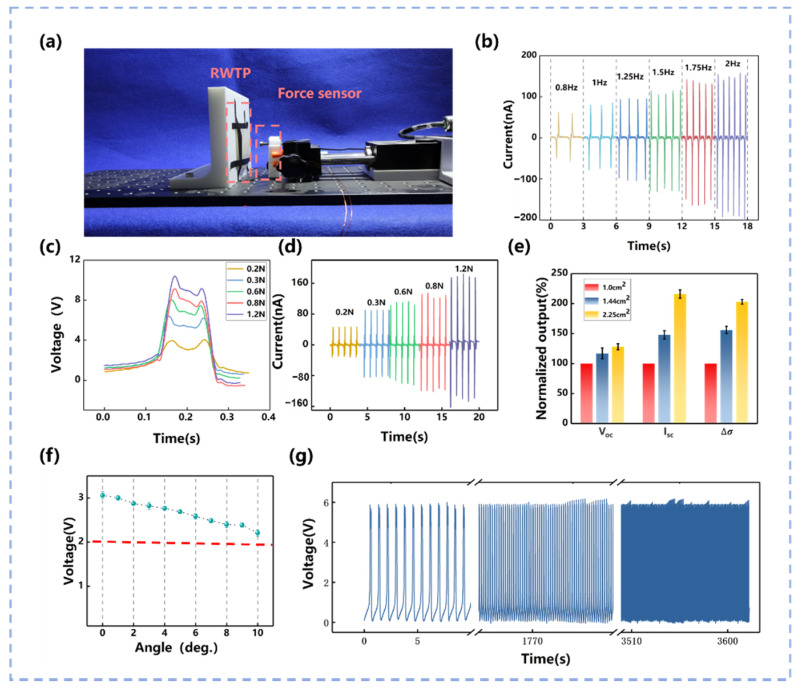
(**a**) Experimental platform with the force sensor and linear motor; (**b**) short-circuit current of RWTP under different frequencies; (**c**) output voltage of RWTP under different forces; (**d**) output current of RWTP under different forces; (**e**) influence of different size of RWTP unit on the output characteristic; (**f**) influence of different force angle of RWTP on the output voltage; (**g**) stability of RWTP.

The electrical performance of the RWTP is shown in Figure 2. Figure 2a displays the experimental platform, which is composed of 3D-printed mold, RWTP, force sensor, and linear motor, where RWTP is fixed on the 3D-printed mold. The end of the linear motor is installed with an 8 mm×8 mm force sensor to detect the force. The force applied to the RWTP is calibrated by repeatedly changing the amplitude and frequency of the linear motor, and display the force applied to the RWTP through the force sensor fixed on the linear motor. RWTP is constantly impacted by the reciprocating movement of the linear motor to obtain the continuous output signals. The output current and voltage signals of RWTP keep consistent and stable. Figure 2b shows the output signals of the short-circuit current (*I_sc_*) at various frequencies of linear motor. *I_sc_* appears to relate linearly with the frequency when it increases from 0.8 to 2 Hz, which shows the quick response and stability of the characteristic of the RWTP. It ensures the practicability of RWTP for further experiments.

When the frequency of the linear motor is 1.25 Hz, the pressure is determined by the amplitude of the linear motor and calibrated by the force sensor. The linear motor applies different pressures to impact the RWTP to obtain a voltage signal. In this way, the force exerted by external motions can be observed. The separation distance *x* of RWTP can be approximated as the deformation of RWTP. The greater the force on the RWTP, the higher the output signal of RWTP can be, as observed in Keithley N6514 (Tektronix, Beijing, China). As shown in Figure 2c, the voltage signal (*V*) effectively reflects different strengths of the external force (*F*); the stronger is *F*, and the higher is *V*. The current signal of RWTP shows the same trend as the voltage signal (see Figure 2d). From the observation above, the RWTP shows a distinguished ability to sense a tiny force compared with the other triboelectric force sensors [45].

**Figure A3:**
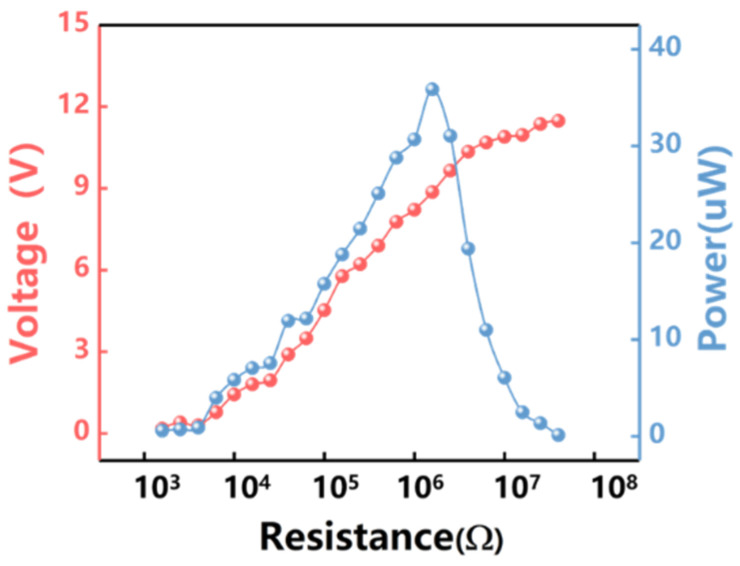
Output voltage and peak power of RWTP versus the resistance of external load.

Considering the different size of hands, the influence of the different size of the RWTP unit on the output characteristics is examined by taking the contact area of RWTP’s dielectric materials at *S* = 1.0, 1.44, and 2.25 cm^2^, and the result is shown in Figure 2e. It is highly evident that the open-circuit voltage (*V_oc_*), short-circuit current (*I_sc_*), and transfer charge ( Δσ ) all increase greatly as *S* increases. For instance, the transfer charge ( Δσ ), which is the most effective parameter for evaluating the output performance, rises by 56% and 103%, when *S* increases from 1.0 to 1.44 cm^2^ and 2.25 cm^2^, respectively. To evaluate the effective output performance of the RWTP, the output voltage is measured with various resistances applied as the external load. The relationship between the output voltage/power and the resistance is plotted in Appendix A Figure A3. Under an external load resistance of 4 MΩ, the maximum peak output power is 35 μW.

Figure 2f demonstrates the effect of different force angle (α) on the output voltage of the device (*V*) at the contact force of *F* = 0.2 N. RWTP is set on the 3D-printed mold (Figure 2a), and the linear motor hits RWTP vertically when the angle is 0°; then, the mold is tilted gradually to change the angle between the linear motor and RWTP. Quite obviously, *V* decreases slightly and approximately linearly with increasing α; when α grows from 0° to 10°, the output voltage of one unit of the RWTP reduces only by 0.82 V. In order to further illustrate the stability of the RWTP, it is impacted by line moter for 3600 s. It is obvious that there is no noticeable change in the voltage signal during continuous impact for 3600 s (Figure 2g).

**Figure 3:**
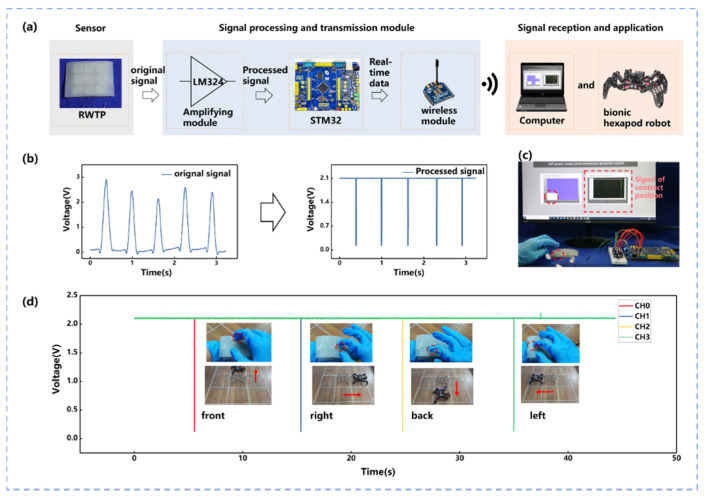
Self-powered contact position distribution detective system and real-time control of hexapod bionic robot based on RWTP. (**a**) Schematic diagram of the contact position sensing system and real-time control of robot based on RWTP. (**b**) Graph of RWTP signal change through signal process circuit. (**c**) Function demonstration of contact position sensing. (**d**) Schematic diagram of controlling the movement of the robot and its corresponding output voltage signal.

A sensor signal reception system was adopted to illustrate the self-powered contact position distribution detective system and real-time control of hexapod bionic robot based on RWTP (Figure 3a).The sensor signal reception system is composed of three parts: sensor, signal processing and transmission module, and signal reception terminal. RWTP was used as a sensor signal generator. The experiment result above illstruates that the RWTP can provide stable signals. In order to improve the resolution of the signals, sensor signals of the RWTP should be processed. A voltage follower circuit built by an operational amplifier chip (LM324) was utilized to improve the resolution of the signals. Since a common amplifier chip has high input and low output resistance, the stability of the output signal can be improved. Once the RWTP voltage signal is beyond the power source (2 V), the output signal can be pulled to 0 V (the deviation is within 0.1 V). Thus, the RWTP can provide a stable signal through processed circuit for controlling the robot, as shown in Figure 3b.The processed signal is transmitted to the signal reception device, such as a computer and robot. When the nine-channel signal is transmitted to the computer, the contact position in RWTP touched by figure can be shown in Labview (Labview 2017), which is a graphical programming software. Figure 3c displays that the RWTP can well perceive the position of touch point in computer by Labview. The location perception of the contact position by finger touch is also shown in Supplementary Materials Movie S1. Processed circuit is built for the RWTP. If multiple fingers press RWTP at the same time, RWTP will generate two signals with almost no time interval between the signals (in Supplementary Materials Movie S1). In fact, RWTP can receive consecutive signals, and it does not need 10 s. Only when RWTP controls the bionic robot to realize the trajectory generation, the input of the trajectory needs to be completed within 10 s as terminator. The robot moves according to the input trajectory after receiving terminator. Compared with other control interfer methods, this processed circuit combined with the RWTP could provide simple and stable signals for robot control. An external force generates a single processed signal [7,43].

It is seen above that the RWTP has an excellent capability of detecting contact position and pressure. Following this, a distinct robotic controlling method using RWTP was designed. The RWTP processed signal is acquired by the ADC converter chip and then transferred to the Microcontroller Unit (STM32F1ZET6). The master control board obtains the processed signal from the MCU by a wireless module (Xbee) and responds to the signal. Through the four channels of the RWTP, the robot is controlled to perform real-time movement in four directions: front, back, left, and right in order to verify the stability of the real-time control of the bionic hexapod robot. Figure 3d shows the CH0–CH3 signals that have a time gap of 10 s, in order to display the signal better. The robot movements in the four directions are also displayed in Figure 3d by touching different channels. The four channels control the hexapod bionic robot to execute the front, rear, left, and right movements, and the corresponding instructions are marked as CH0–CH3. The bionic hexapod robot real-time motion controlled by the RWTP is shown in Supplementary Materials Movie S2.

**Figure A4:**
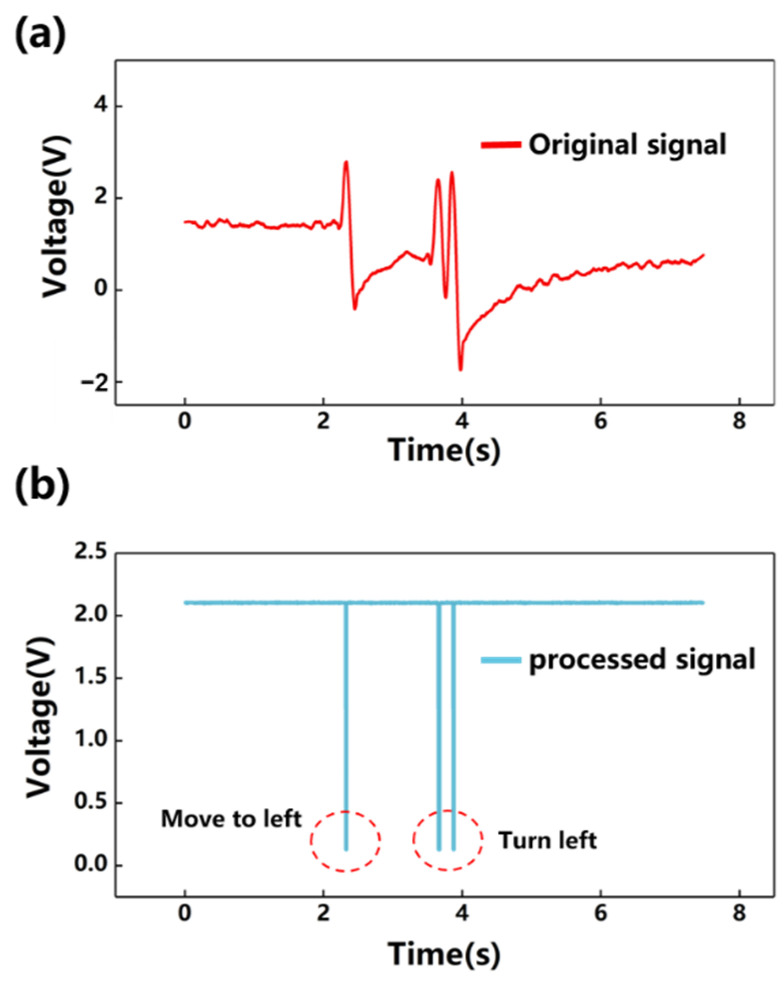
Transferred signal of the RWTP: (**a**) original signal and (**b**) processed signal.

Further, to enrich the motion posture of the bionic hexapod robot, a channel is also set to control different actions. The specific method is to complete different controls by clicking the same channel multiple times within 2 s. When one of the channels receives a signal, the bionic hexapod robot immediately executes the movement instruction. As shown in Appendix A Figure A4, Channel 3 is clicked once to let the robot move to the left, and twice to turn to the left. In this way, the control breadth of the RWTP can be increased, which allows the robot to move freely in two-dimensional space. It is expected that the robot will be even controlled to move freely in three-dimensional space when the present device is further advanced in the future.

**Figure 4:**
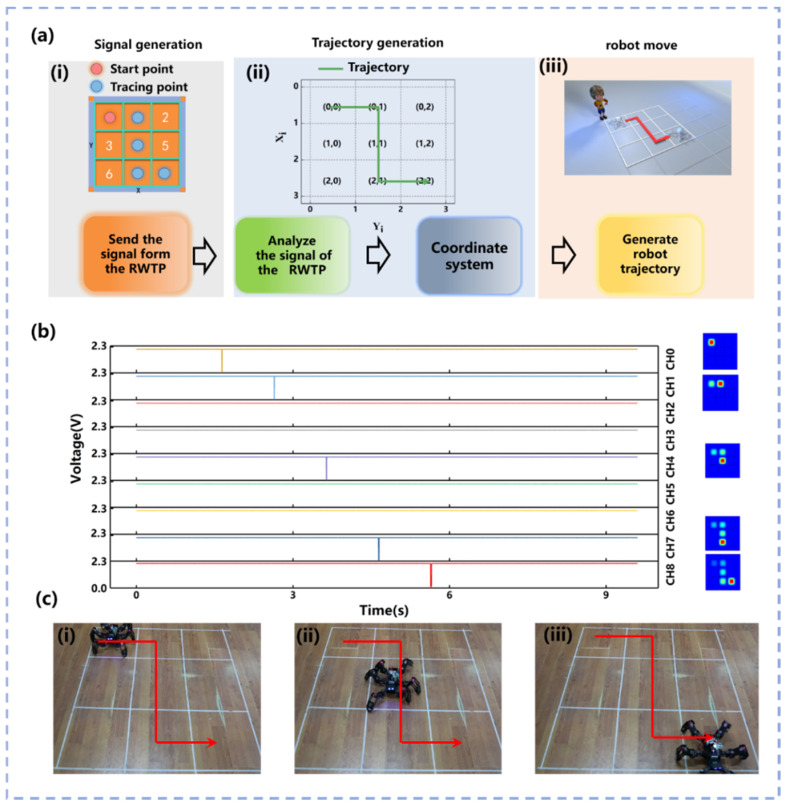
Demonstration of robot trajectory generation. (**a**) Logic diagram of trajectory generation based on RWTP for robot control. (**b**) Schematic diagram of the signal when the RWTP controlled the bionic hexapod robot to generate a trajectory. (**c**) Schematic diagram of bionic hexapod robot trajectory generation.

As shown in Figure 4a(i), the 3 × 3 RWTP unit can communicate with the host computer through nine channels of the circuit and the MCU in the above experiments. Therefore, the trajectory generation is achieved through utilizing the nine units of RWTP completely. As shown in Figure 4a(ii), the trajectory generation of the bionic hexapod robot is achieved by slidings finger on the RWTP. The bionic hexapod robot can be connected with RWTP by constructing the 2D coordinates system through Equation (6).

(6) $$\{\begin{array}{c} i=\lfloor LOC\left(a_{i,j}\right)÷L\rfloor \\ j=LOC\left(a_{i,j}\right)-i\times L \end{array}$$

In Equation (6), *i* and *j* are the *X* the *Y* coordinates; $LOC\left(a_{i,j}\right)$ is the serial number of the RWTP, where the nine channels of RWTP are numbered 0–8; and *L* is the number of dimension of the coordinate system, where is 3 in our system. RWTP serial number ( $LOC\left(a_{i,j}\right)$ ) is divided by *L*, the quotient is the *x*-axis coordinate, and the *y*-axis coordinate equate to the *j*, so that the 1D coordinate can be converted to the 2D coordinate system. The working principle is as follows: each position of the bionic hexapod robot is associated with the two-dimensional coordinates that vary at the same time when the position of the bionic hexapod robot changes. The bionic hexapod robot movement can be determined through the variations of the x and y coordinates, e.g., the position (1, 1) to (1, 2). This means that the coordinate x has not changed, but y has, so the trajectory of the bionic hexapod robot only varies along the *y*-axis. Figure 4a(iii) displays the demonstration of the actual trajectory generation. The robot is placed on the constructed two-dimensional plane and connected with the matrix of the RWTP. In order to determine the starting position, the unit point where the finger first touches the RWTP is (0, 0). The finger touches the units in an appropriate order that make the bionic hexapod robot to walk along the preset trajectory. Commands are transmitted to the hexapod robot through the wireless module, which can make the robot generate a trajectory as planned.

Figure 4b illustrates a schematic diagram of the signal when the RWTP controlled the bionic hexapod robot to generate a trajectory. Unit 1 is regarded as the starting point to make the bionic hexapod robot to move according to the trajectory of Figure 4a(ii). Each unit will not interfere with each other, since it is independent. The bionic hexapod robot accepts the series of signals, makes judgments through the abovementioned logic, and moves according to the preset trajectory on the RWTP after units 1, 4, 7, and 8 are touched one by one.

The signal diagram received by the nine channels of the MCU and electric potential distributions for the RWTP units through simulation software COMSOL are shown in Figure 4b for when the bionic hexapod robot moves according to the preset trajectory of Figure 4a(ii). Since the bionic hexapod robot needs to judge the input trajectory signals, the trajectory generation cannot be considered as a real-time control method. After a series of commands from MCU are received by the robot within a period of time set by actual operating requirements, the robot starts timing when one of the nine channels of MCU first receives and then executes the movement instruction after the preset time period passed. When RWTP controls the robot to realize the trajectory generation, the input of the trajectory needs to be completed within 10 s as terminator. The robot moves according to the input trajectory after receiving terminator. The trajectory of the robot movement is shown in Figure 4c(i–iii). The robot can execute different trajectory commands according to different touches’ order of units. The corresponding trajectory generation diagram is shown in Supplementary Materials Movie S3.

## 3. Conclusions

In this work, we developed a self-powered TENG-based minimalist interactive and more effective interface for sensing and controlling robots. First, hydrogels were used as the flexible electrodes to transfer the external force into the deformation of RWTP. Second, this paper proposed the signal processing circuit which can convert the sensor signal into a more stable signal; the deviation is within 0.1 V, which provides a more effective method for sensing and robot control. Third, the intermittent contact motion of the finger can be converted into trajectory information. Thus, a robust and reliable detection method can be established through the output signals of nine RWTP units.

Therefore, the sensing information of the finger touch (such as contact position, sliding track, applied pressure, etc.) can be detected by the minimalist interactive patch of 3 × 3 units. Theoretically, the minimalist patch (RWTP) with greater stability will further achieve a higher resolution. The RWTP can be used to control the movement of robots in real time and performs well for its high resolution and sensitivity. The trajectory of a bionic hexapod robot can be recorded through the RWTP and a fitted nine-channel signal acquisition-processing circuit. Therefore, the 1D serial number can be converted into a 2D coordinate system, so that the click of the finger can be converted into a sliding track. Accordingly, the human–machine interaction device-based RWTP with minimalist design and flexible materials will have a great application potential in pressure sensors, 2D control, robotics, and wearable electronics.

## 4. Methods

### 4.1. Signal Acquisition Circuit and Signal Processing Design

The signal acquisition circuit was built by using three LM324 chips, STM32F1ZET6, and a set of Xbees. The LM324 chip was used as a signal-processing circuit and supplied by the 2 V power. When there is no signal at the input port, the output signal of the LM324 is 2 V. The signal generated by RWTP is pulled down through the LM324 chip circuit to get the output signal. The output signal is connected to the 9 I/O of the STM32F1ZET6 single-chip microcomputer board. The two Xbees are connected through serial communication in order to control the robot. The instructions of the four steps are controlled by the four units of RWTP. The bionic hexapod robot is equipped with an Arduino Master control board and a wireless module to receive control signals from the STM32F1ZET6.

### 4.2. Fabrication of Device

The silicone rubber was prepared with A and B silicone rubber solution with a shore hardness of 30, A and B silicone rubber solution were mixed in a ratio of 1:1, and poured into the 3D-printed mold. There are two types of molds, one is 7 × 7 cm, with a height of 1 mm, and it is used as the lower silicone base mold. The other is 10 × 7 cm, with a height of 1 mm. A single unit of 1.5 × 1.5 cm, with a height of 2 mm, was arranged in a 3 × 3 manner in this mold with 1 mm distance between the individual units. This mold was used as the upper silicone base mold. The silicone was poured into the mold and was left to stand still for 24 h, at room temperature, and after that, the silicone was taken out as a silicone rubber substrate. After that, the hydrogel electrodes were attached to nylon and PTFE, and then connected to the silicone rubber substrate. Finally, RWTP is composed of silica rubber substrate, hydrogels electrode, nylon PTFE hydrogels electrode and silica rubber substrate from top to bottom. In order to make sure that the RWTP has a stable output performance, we packaged the device with waterproof PTFE tape. The triboelectric layers were enclosed by PTFE tape. The triboelectric layers were isolated from the air; the triboelectrification performance of RWTP cannot be affected by the relative humidity of the air.

### 4.3. Characterization and Electrical Measurements

Models used in the COMSOL simulation software are AC/DC modules. The microstructure of nylon and PTFE was photographed with an optical microscope (LEXT OL-S4000, Olympus, Shenzhen, China). Open-circuit voltage and short-circuit current were measured with Keithley electrometer system (Keithley 6514, Tektronix, Beijing, China). The sensitivity to pressure of the test device is calibrated by a force sensor (LZ-ZY2, Lizhi, Hefei, China) and a linear motor.

## Data Availability

Data available on request due to restrictions privacy. The data presented in this study are available on request from the corresponding author. The data are not publicly available due to involving professional technology.

## Supplementary Materials

The following are available online at https://www.mdpi.com/1996-1944/14/21/6366/s1. Movie S1: RWTP for sensing touch point. Movie S2: RWTP for controlling robot real-time. Movie S3: RWTP for trajectory generation of the robot.
